# Proof of Concept Novel Configurable Chipless RFID Strain Sensor

**DOI:** 10.3390/s21186224

**Published:** 2021-09-16

**Authors:** Kevin Mc Gee, Prince Anandarajah, David Collins

**Affiliations:** 1 School of Biotechnology, Dublin City University, D09 NRT0 Dublin, Ireland; david.collins@dcu.ie; 2 The National Centre for Sensor Research (NCSR), Research & Engineering Building, Dublin City University, D09 NRT0 Dublin, Ireland; 3 Photonics Systems and Sensing Laboratory, School of Electronic Engineering, Dublin City University, D09 NRT0 Dublin, Ireland; prince.anandarajah@dcu.ie

**Keywords:** chipless RFID sensor, strain sensor, chipless RFID tag

## Abstract

This paper contains two main areas of research: First, this work outlines a novel, highly sensitive strain sensor design that should support various levels of deformation, depending on the substrate type used. Physical implementations in this work have focused on proving its large deformation capabilities, and simulations have been used to assess its more general electromagnetic response. The other part of this paper focusses on exploring other effects that will impact the sensing of strain of resolutions below 10 με, which is a capability achieved by other aerospace-grade strain sensor technologies. These effects are limited to mechanical swelling and sensor orientation in the azimuth and elevation planes, as these appear to be unexplored and highly relevant issues to the topic of chipless RFID-based strain sensing. From this exploration, it is apparent that the effects of mechanical swelling and sensor orientation (amongst others) will need to be addressed in any real-life implementation of the sensor, requiring a strain resolution below 10 με.

## 1. Introduction

### 1.1. Introduction

**Figure 1:**
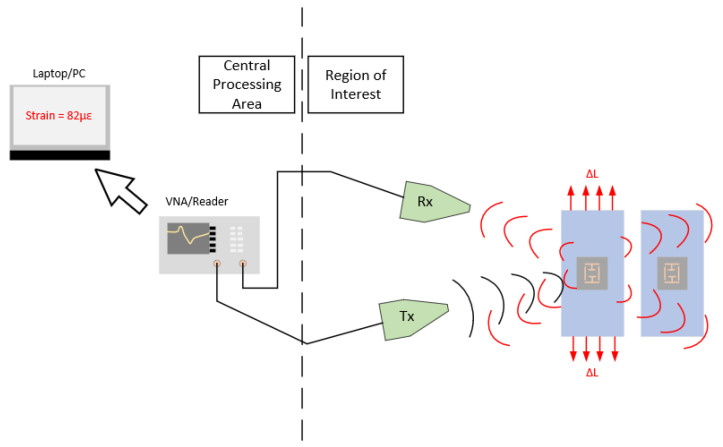
Envisaged system diagram.

Chipless RFID sensor research is quickly becoming an area of great interest in modern power-aware application spaces. Thus far, a great deal of work has been conducted in the development of strain sensing chipless RFID sensors and many works have reviewed this body of literature in detail, including one by the authors in [1]. The envisaged strain sensing solution includes the developed sensor(s) attached to a structure of interest. A specially designed reader system would interrogate the tags in a monostatic or bistatic configuration with directive antennas that support beam steering capabilities. This envisaged reader system would readily support successful detection of resonant signatures in a variety of real-world environments. Such a system may not exist currently, but it is assumed that future reader systems could support such functionality. Finally, the datasets generated by the reader are sent to a central controller/computer for analysis, interpretation and storage. Figure 1 gives a brief overview of the envisaged system. In terms of sensor fabrication, the idealized device should be fully capable of being rapidly printed in-situ using existing direct technologies, that are controlled in an automated way.

**Figure 2:**
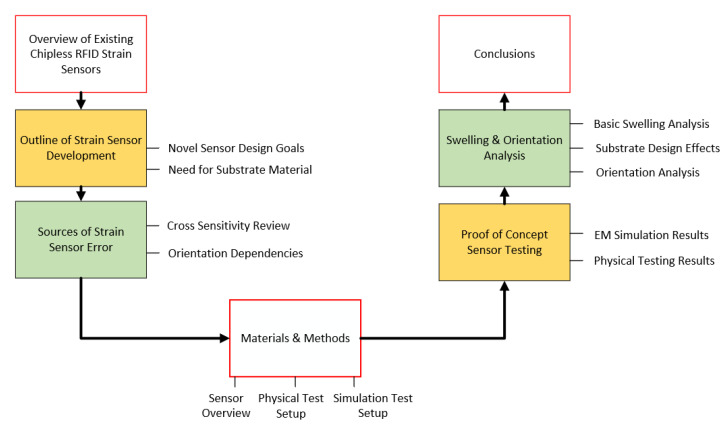
Diagrammatic layout of Paper 1.1. outline of strain sensor development.

This work attempts to add to the current state-of-the-art chipless RFID sensor design with a novel sensor design that, in its current instantiation, exhibits a gauge factor of 1.56. A more general approach is taken in this work towards the design of the strain sensor, so that the current resonator design could be used in a variety of different strain sensing applications. This means that emphasis was put on developing a resonator design that could be made to support an arbitrary range of strains through the selection of an appropriate substrate material. Applications for sensors of this type include aerospace structures such as those outlined in [2,3]. These applications and others will require varying strain sensing requirements. One of these requirements is minimum stimulus range, which may be as low as 0.2% or higher than 10% [4]. This paper contains two main avenues of exploration that are relevant to the development of a suitable, extreme environment-suitable chipless RFID strain sensor. These are the outline of a novel strain sensor design and an exploration of overlooked causes of chipless RFID strain sensing error, namely the effects of swelling and the effects of sensor orientation. Figure 2 presents a graphical depiction of the contents of the paper.

#### 1.1.1. Review of Existing and Proposed Sensor Designs

The developed strain sensor is based on the Electric-LC (ELC) resonator used in various metamaterial related publications. This resonator was considered as a base resonator through which strain sensing could be achieved as it exhibits a small footprint, and it has a large central capacitance which other works have utilissed in successful sensor designs [5,6]. From the existing literature on chipless RFID strain sensor design, it can be seen that the resonators developed make use of a variety of different deformation mechanisms to alter their resonant responses. These include elastic deformation [7,8], bending [9,10] and to some extent, rigid body motion [7]. The latter occurs when the substrate expands between conductive regions to result in a change of capacitance or a change in capacitive/inductive coupling. Another observation of the current body of literature in this area is that the use of elastic deformation or bending as the primary sensing method pushes a great deal of emphasis on the mechanical properties of the deposited conductors. A successful design of great note in this particular conversation was created by Min et al. in [11], which makes use of an AgNP/MWCNT-based deposition that supports strain levels over 20%. Other works such as that by Teng et al. in [9] rely largely on the bending of an MLA antenna and support strain levels of up to 50%. The latter work made use of the liquid metal Gallinstan to support these strain levels. Other designs have focused on the sensing of strain levels below 10000 με (1%) such as those referenced in [7,12,13,14,15]. Works of note include those by Thai et al. in [14,15] which make use of cantilever mechanisms to develop highly sensitive strain sensor designs. Although very impressive strain sensitivities are achieved with these works, they require the fabrication of a suspended cantilever which brings significant fabrication complexity to the implementation of these designs with direct write technologies. The work of Chuang, Thomson, and Bridges in [16] and other works have developed strain-sensitive resonant cavity designs that can be interrogated with RF frequencies. The designs and analysis presented in this work, however, have focused on a planar design so that it can be readily deposited in situ using techniques such as inkjet or aerosol deposition.

Of importance to this discussion is that many of the aforementioned sensor designs make use of different resonator types and different substrate materials and operate at different frequencies. Some works have attempted to compare these various strain gauge designs using metrics such as gauge factor, maximum range, and many other metrics to compare the various chipless RFID strain gauge designs. However, it would appear that such comparisons do not reveal the optimal chipless RFID strain sensor design. This opinion is put forward in this paper since it does not seem to be possible to compare strain sensors that operate over a variety of different strain ranges due to the fact that it is not necessarily the case that each resonator design (SRR, ELC, MLA) is capable of achieving an arbitrary strain sensitivity and range. In general, it would appear that the choice of substrate material and its height will dictate the general performance of the strain sensor and that the relative mechanical properties of the conductor and substrate will dictate the dominant deformation mechanism within the resonator. One observation of the existing RFID sensor literature is that sensors that exhibit multiple deformation mechanisms seem to exhibit more impressive performance compared to the others and may more readily support a large variation in stimulus range that could be tailored simply through the changing of substrate materials. This observation is made based on the fact that large strain levels (>20%) would require a highly tailored substrate to convert that strain to a level suitable for a strain resonator that operated purely via elastic deformation (<0.5%).

#### 1.1.2. The Use of a Dedicated Substrate Material

The next point to be made in this section is to highlight the advantages of using a dedicated substrate material which sits between the MUT and the resonator. Largely speaking, many dielectric strain sensing applications could avoid the need for such an addition but there are advantages of including a known dielectric material onto which the resonator is applied. These advantages include:

- Sensing of strain on metallic or general conducting materials will require an intermediate material between the MUT and the resonator;
- It would be advantageous to have a consistent resonant response location in the RCS response which the use of a dedicated substrate would help achieve, as the dielectric MUT may have a significantly different permittivity [17];
- Certain dielectric materials have significant loss tangents [17] and the use of an intermediate dielectric could help mitigate its detrimental effects on the resonant response of the sensor;
- The strain performance of the sensor (sensitivity and range) can be tuned via the use of a specific substrate material and height;
- Significant levels of surface roughness and curvature of the MUT may cause difficulties in successfully/accurately depositing the resonator in place. A substrate material could help provide a smooth, flat surface for conductor deposition;

The disadvantages of using a dedicated substrate material include:

- This material could negatively impact the ability of the strain sensor to function. Examples of how this may occur include the effects of substrate swelling. This will become an issue of particular interest if the expansion coefficients of this material differ than that of the MUT, which is most likely going to be the case;
- Certain materials may readily absorb the strain induced within their bottom surface by the MUT and not successfully impart this deformation to the resonator on their top surface. This will most likely only be a concern for flexible substrate materials such as soft rubbers when the MUT is under low levels of strain. Although the substrate height can be altered, there will be limitations on the thickness resolution of easily deposited thin films;
- The choice of substrate material may not be freely within the sensor designer’s choice as the environment that the sensor will be used in may dictate the use of unfavorable materials;

The conclusive point from this discussion is that there are many benefits to the use of a dedicated substrate material, some of which necessitate its use, i.e., strain sensing of electrically conductive MUTs. Similarly, it would seem that no one substrate is suitable for all strain sensing applications and that other substrate induced effects may hamper sensor performance. Overall, a dedicated substrate will be needed for sensing on metals and, since the proof strain of metals is around 0.2%, that substrate will most likely need to be sufficiently stiff to impart those strains onto the resonator. Similarly, other applications will require a larger strain range and will require a more flexible substrate. Therefore, the best way to support all of these possible scenarios is to develop a sensor design that can operate under all of these conditions.

#### 1.1.3. Novel Sensor Design Goals

The main goal of this work is to develop a basic chipless RFID resonator that should be suitable for an arbitrary strain sensing application, whether it needs to support a maximum stimulus range of 0.1% or 100%. Furthermore, this design should not force the choice of conductor to extreme criteria, such that only a select few materials can meet. This comment is made as materials such as liquid metals are sensitive to temperature levels that may be encountered in aerospace settings [18,19]. Ideally, the design of this sensor should also support its fabrication with direct-write technologies.

To support various applications that require the sensing of strain within a large range, this resonator design will focus on using rigid body motion as the main deformation mechanism when being used with a flexible substrate. This design should also support bending and elastic deformation mechanisms to allow for its use on stiffer substrates, so that it is capable of high performances for low strain level (<0.X%) sensing. Thus, the implicit goal of the design is to support operation on a variety of substrate materials, whether they are a soft rubber or are a rigid epoxy material.

### 1.2. Sources of Strain Sensor Error

As many aerospace strain gauge applications may require strain sensing resolution on the order of 10 με [4], the effects of other stimuli may become the dominant contributor to the sensor response [16]. This section reviews the sources of error/accuracy that may arise in chipless RFID strain sensors, that falls under the umbrella of sensor performance.

#### 1.2.1. Cross-Sensitivity Issues in Chipless RFID Strain Sensors

The following table (Table 1) describes the general effects that will intuitively affect the resonant response of the chipless RFID sensor and the material behaviours that may give rise to said effect. Comments are not made as to the change in resonant response to be expected from each effect as differing resonant behaviours may occur depending on the resonator design.

This paper will explore the effects of conductor and polyimide substrate expansion as these are mechanical effects which are largely ignored in the existing chipless RFID literature and due to the fact that the previous section outlined scenarios where a dedicated substrate will be required, which may give rise to swelling-induced sensor errors. These mechanical effects are demonstrated in this work to present a significant challenge to the sensing of strain within 10 με. 

Polyimide is a strong, high temperature polymer that has been used in aerospace settings previously and is a material of great interest in the area of printed electronics. Furthermore, polyimide and other polymeric materials have been deposited in-situ previously [28,29], which would make them possible candidate materials for the development of a fully printable chipless RFID strain gauge. The authors of this work are interested in using this material as the dedicated substrate for the sensing of strain below 0.2%. The effects of swelling were not successfully mitigated against in this work and thus it is the conclusion of the authors that these problems will need to be compensated for via reference resonators or by an alternative approach.

#### 1.2.2. General Orientation Issues in Chipless RFID Sensing

The other source of error explored in this paper is the interrogation challenges presented by this resonator design and that of a regular ELC resonator. The reason why this issue is being investigated before others is that the envisaged system in Figure 1 will consist of multiple chipless RFID sensors and a single or very few reader antennas. Therefore, some, if not all of sensors will not be orientated perpendicular to the boresight direction of these antennas. Of interest to this work is the effect of the interrogation angle in the azimuth and elevation planes on the measured resonant response of the sensor, assuming no polarization mismatches occur. Possible solutions to issues such as polarization have been outlined in other works such as [30,31,32,33,34,35] and avid readers are directed to those publications for an in-depth discussion on the challenges and solutions to polarization mismatches. The reasons why polarization mismatch is neglected in this work is that the desired strain gauge design should be sensitive in only one direction and that many of the polarization-independent resonator designs do not appear to readily allow themselves to becoming invariant to transverse strain. 

The general issue of small changes in resonant response may not be of critical importance in chipless RFID addressing as small deviations in the resonant location in the frequency response may be of little importance. This is not the case for sensing applications and several works [36,37] have described orientation-dependent properties of the resonator response and its possible connection to the 3D RCS of the tag. Avid readers are pointed towards the work of Alam et al. in [37] which peak and null radiation patterns are presented for their chipless RFID tag design which clearly show the presence of side lobes in the null response that are not present in the peak response. In such a design, it is inevitable that these side lobes will cause the measured resonant signature to vary depending on the orientation angle of the reader with respect to the tag. The results presented in this work depict this dependence. These and the results of other researchers [36,37] would suggest that it is more likely than not that many REP-based chipless RFID tags/sensors will exhibit orientation dependent responses.

## 2. Materials and Methods

**Figure 3:**
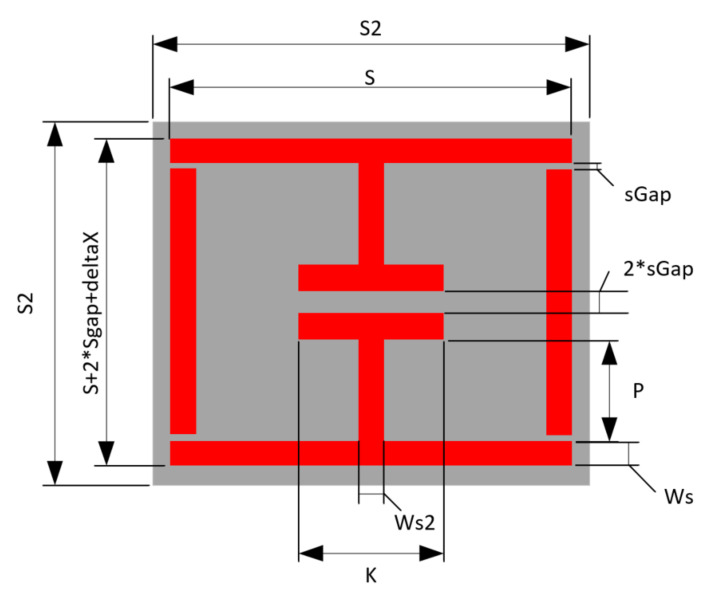
Labelled sensor diagram.

The overall sensor design is depicted in graphical form in Figure 3. The design makes use of rigid body motion between the side walls (EL parts) and the upper and lower parts (EC parts). The variable “sGap” depicts the distance between these parts. Several different implementations of this sensor were developed; some used conductors cut out (EDM) from copper sheet and the others make use of a cheap commercial conductive ink (RS: 123-9911) [38]. 

### 2.1. Sensor Implementation

**Figure 4:**
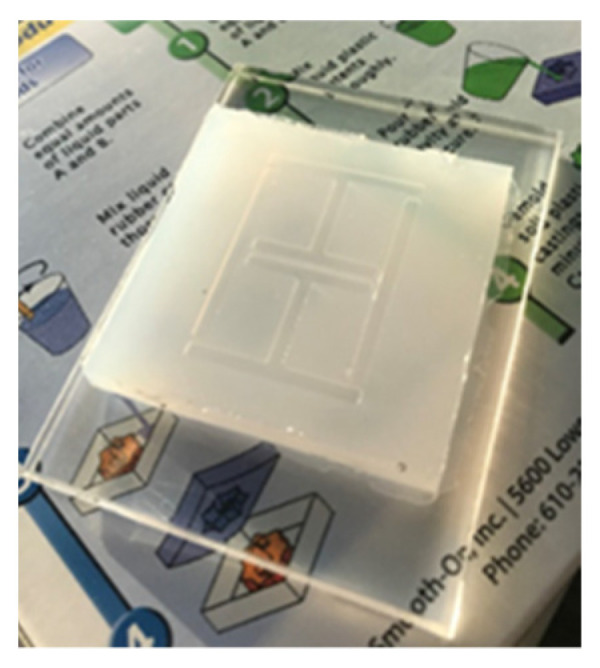
Ecoflex substrate.

Several sensor implementations were fabricated during this work. A basic sensor was made using cheap commercial ink as a conductor material and used Ecoflex silicone rubber [39] as a substrate. Table 2 depicts the geometries of this implementations. Adhesion between the conductor and silicone substrates were achieved via submersion of the conductor inside the substrate, as the cured silicone surface was too difficult to adhere to without the use of complex surface treatments. At this point, it must be highlighted that the use of more expensive conductive inks and fabrication processes may allow for printed conductors that are more mechanically robust, but this work avoided these needs through the use of additional supporting materials. The Ecoflex silicone rubber substrates that were going to be used with the cheap ink would have trenches moulded into them (see Figure 4) to allow for the inclusion of a stiffer polymer material to sit under the conductor. This inclusion also avoided the need for surface treatment of the silicone rubber before conductor deposition. Inclusion elements were made from various plastics that were laser cut into shape and other implementations used polyester resin (ISOPON FASTGLAS) [40] deposited into the trenches using a screen-printing procedure. The cheap conductive ink was painted in place with an artist’s brush. The performance of various filler materials is not reviewed in this work but the success of using a polyester resin as a filler may allow for successful in-situ fabrication of this part of the sensor.

**Figure 5:**
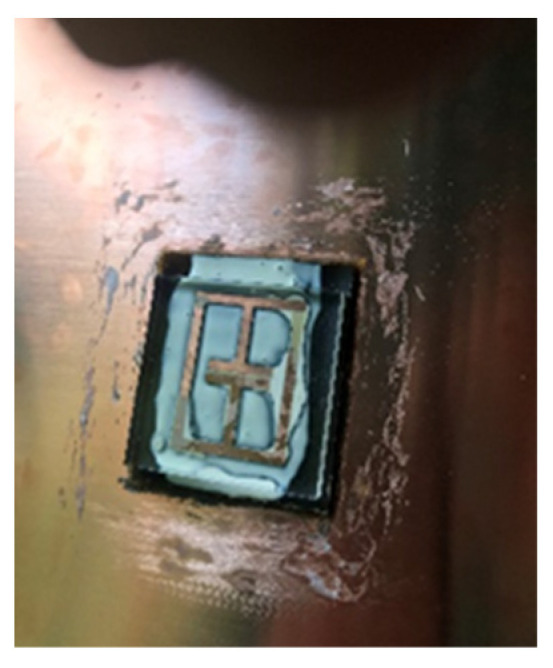
EDM latex tag on copper sheet.

**Figure 6:**
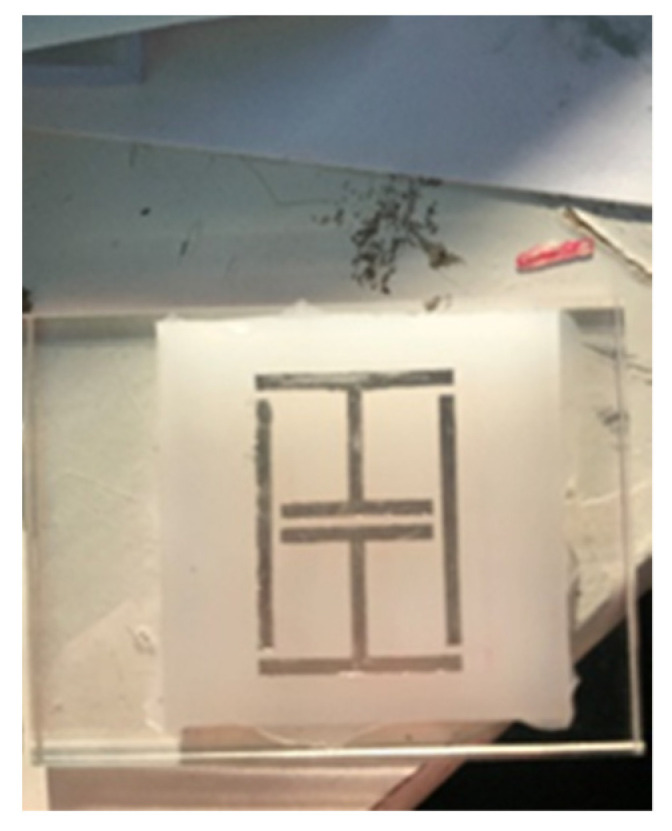
Finished Ecoflex tag with conductive ink.

Other implementations used thicker conductors that were cut out of 0.3 mm thick copper sheet, using an EDM process. These implementations use liquid latex rubber (see Figure 5) and silicone rubber substrates (see Figure 6). Table 3 and Table 4 depict the geometric characteristics of these sensor implementations.

### 2.2. Test Setup

**Figure 7:**
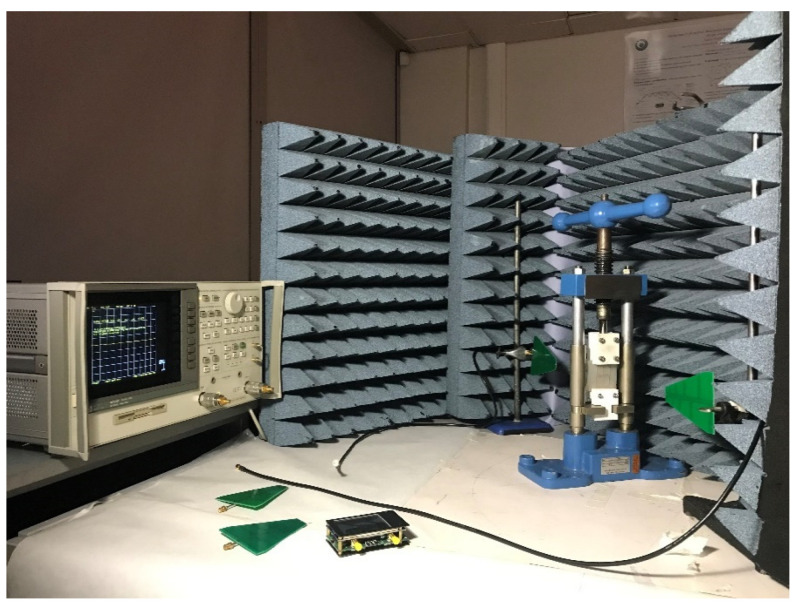
Opened test environment.

The various sensor implementations were tested in a small anechoic chamber using a HP8753D and NanoVNA V2_2 Vector Network Analysers. The majority of strain sensor performance testing was performed with the bench press located between the transmitting and receiving antennas, each separated from the sensor by 25 cm. The strain testing performed in this work exclusively deformed the substrate material of the sensor, via the use of 3D printed plastic clamps. In more idealistic testing, the substrate would have to be be adhered to a superstrate and that part would be then deformed. Log-Periodic Dipole antennas were used for transmission/reception and have a gain of between 5–6 dB over the relevant frequency range. Basic testing of the sensor on metallic superstrates involved repositioning both antennae to face the exposed side of the sensor. Transmission power was limited to 0 dBm during all testing and results were consistnely within the dynamic range of the VNA. Figure 7 depicts the opened/exposed test environment. With regard to the performance of these and other chipless RFID sensors in realistic environments, the authors would like to point out that background subtraction cannot counteract all multipath effects in an environment as it cannot be certain that sufficient power reaches the sensor in an arbitrary setting. Furthermore, these sensors do not exhibit an idealistically directive RCS response and thus the return path could also suffer from significant multipath degradation. Readers interested in further work aimed at combatting multipath effects are directed to the work of Megahed in [41]. Likewise, this work does not explore the response of this tag using different various reader types or explore the response of the sensor to the effects of vibration. The effects of a dynamic stimulus have been discussed in [42] and more general response characterization from time-domain based readers can be found in [43] by Babaeian and Karmakar and by Kalansuriya et al. in [44].

### 2.3. Modelling of Sensor

Ansys Campus-based FEM software is used to perform the relevant sensor simulations in this work. Ansys HFSS [45] is used to model the electromagnetic behaviour of this device and Ansys Mechanical is used to perform the relevant steady-state structural and thermal/humidity analysis. The former simulation environment includes all of the relevant material properties for EM simulation and other parameters needed for mechanical modelling are taken from the relevant published literature. The HFSS environment makes use of a built-in meshing system that iteratively increases the mesh resolutions such that the results at a specific (meshing) frequency converges within a certain deviation between successive mesh iterations. A plane wave excitation was used at a distance of 10 cm from the sensor and bistatic RCS results were used to explore the orientation dependency of the null location. To simulate the effects of a metallic superstrate, a perfect electric conductor (PEC) boundary condition was used. 

This sensor design is meant to support different substrate types, so that the sensitivity and range of the sensor can be tailored. To this end, Ansys Mechanical [46] FEA modelling has been performed to assess the degree to which the different deformation mechanisms (expansion, bending, rigid body motion) occur during loading of various types. A general introduction to the FE method can be found in [47]. The results in this paper focus on the use of a polyimide substrate, as it is a highly inert material that has a known track record in aerospace applications and is used extensively in the printed electronics industry. Simulation results related to the use of Ecoflex silicone rubber have been omitted as they do not significantly contribute to the discussion at hand. The behaviour of the sensor with a stiffer substrate was a more pertinent aspect to investigate for the following reasons:

- The physical test results have clearly proven the strain sensing ability of this resonator when used with soft substrate materials;
- As polyimide is much stiffer than rubber, the degree to which rigid body motion will occur in the operation of the sensor will undoubtably be reduced. Therefore, it is important to assess what contribution each deformation mechanism makes in the sensor operation;
- Stiffer substrates may benefit from additional, novel substrate modifications such as slots, etc. so that device sensitivity can be more specifically tailored;
- The performance of polyimides in aerospace settings has been well characterized and their cross-sensitivities have been explored extensively in literature.

These simulations have assumed that both the conductor and substrate have a linear elastic stress response and, thus, more complex effects such as creep have been neglected. Such an assumption has been made about polyimide materials based on the stress-strain relationship reported in [48,49]. The material properties relevant to this discussion can be found in Table 5. Also, all of these material parameters may differ significantly depending on the fabrication method, materials and the fabrication environment. Such variations may be isotropic or anisotropic [50,51] in nature. Another point of note is that this analysis has no temporal component but in reality, the substrate and conductive materials would not conduct/radiate or absorb/release temperature or moisture instantaneously.

**Figure 8:**
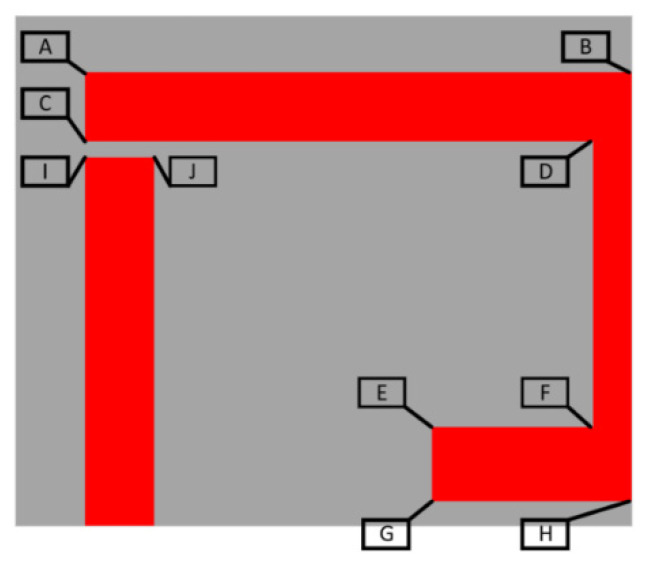
Labelled diagram for FE analysis.

**Figure 9:**
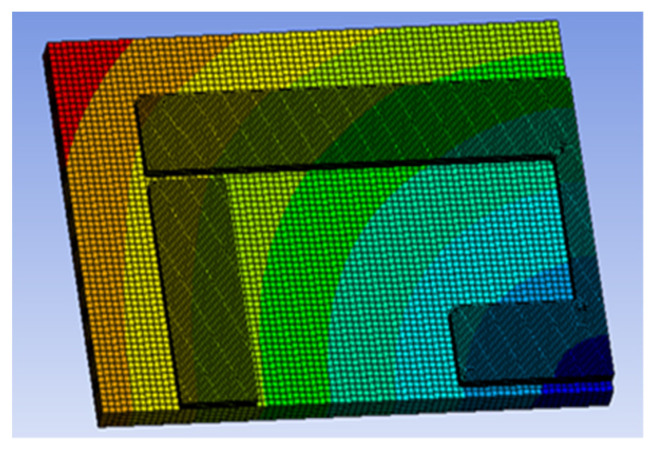
FE model with deformation contour plot (images courtesy of ANSYS Inc., Osaka, Janpan).

Most of the simulation results presented in this work focus on the effects of material swelling on the mechanical behaviour of the device. The geometry used to evaluate mechanical expansion included a thin substrate (500 μm) as opposed to the larger ones of the physical sensor implementations, as earlier testing revealed that the deformation of the top surface increases significantly with increasing substrate thickness. A slightly different version of the sensor design was used for this analysis and the dimensions of this device are given in Table 6. These include thermal expansion and humidity-based expansion. The latter was simulated through the use of the equivalent expansion coefficient for humidity, which is referred to as the coefficient of hygroscopic expansion (CHE). Polyimides can have anisotropic CHE values in ranges including that of 60 ppm [25] to 90 ppm [20] and this work used 90 ppm as the default value. The effects of humidity on copper were neglected as although degradation may occur, these effects occur on a much larger time scale than substrate swelling. Figure 8 depicts the labelling strategy used to characterise the resonator response and Figure 9 depicts the simulation model and converged mesh used to represent the sensor. This latter figure includes an overlaid contour plot of the total thermal deformation of the sensor under 350 °C change in temperature. As the design exhibits two planes of symmetry, only one quarter of the model needs to be simulated and symmetry or free planes can be used to represent the other sides of the sensor. The bottom of the substrate is set in place with a free plane for the axial testing and with a fixed plane for the majority of the swelling tests. The latter was chosen so as to represent a superstrate that is not experiencing any form of deformation, whether it result from external forces or thermal/humidity-based swelling. Bonded contact regions were used between the conductor and substrate parts. Axial deformation was applied to the external face of the substrate with a magnitude of 0.05 mm which corresponds to a strain of approximately 0.33%.

The convergence of the results of the axial deformation tests was achieved through iteratively increasing the element count in each part of the design. As thermal expansion led to comparable deformation levels when used with the same boundary conditions as the axial deformation, it was found that increasing mesh resolution under this loading strategy did not alter the results of these tests significantly. Thus, the same mesh was used throughout all of the analysis.

**Figure 10:**
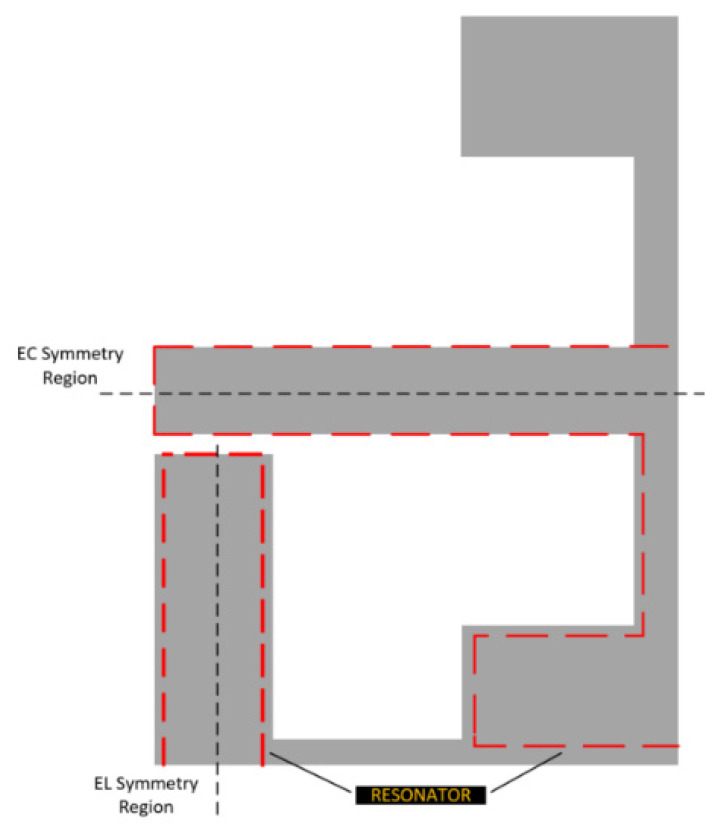
Symmetry-based substrate design.

**Figure 11:**
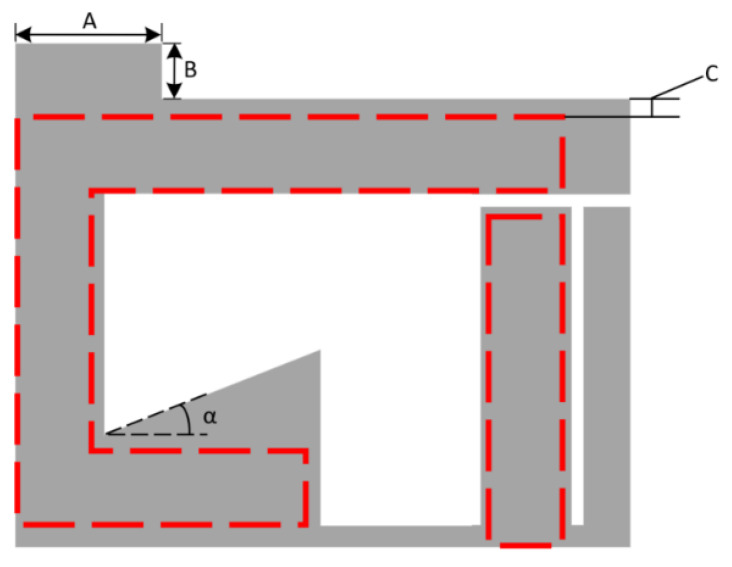
Tuneable substrate design.

The next question to be asked is whether these effects can be compensated for within the design. Early attempts made use of copper guard rings that would constrain the substrate, which could be deposited in situ with the resonator. These and other attempts were unsuccessful, and the next method explored was the modification of the substrate geometry. This paper reviews attempts at the alteration of the substrate design such that the effects of material swelling could be minimised. The two substrate designs used can be seen in Figure 10 and Figure 11. The first design makes extensive use of symmetry as it was believed that this may reduce the axial rigid body motion deformation effect within the strain sensor. This was considered to be the case as swelling-based deformation of any free body will be zero in any chosen direction at a central/centroidal point in that particular body. The other design attempts to build on this idea such that the main capacitances of the design are not affected by swelling. This design involves the iterative modification of the various dimensions labelled in Figure 11 such that the deformations at the key points of interest are mitigated.

## 3. Results and Discussion

### 3.1. Proof-of-Concept Sensor Testing

#### 3.1.1. Electromagnetic Simulation Results

**Figure 12:**
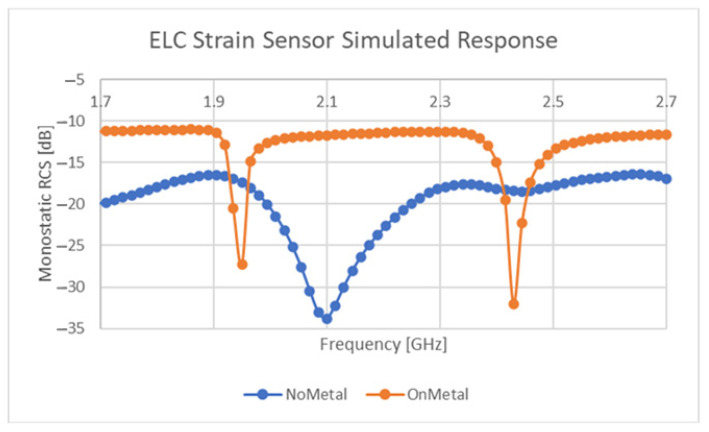
Simulated sensor response.

The response of the sensor design outlined in Table 2 with a polyimide substrate material can be seen in Figure 12. This response includes the response of this design both with and without a metallic superstrate. These simulations and physical testing revealed that this resonator appears to exhibit a separate resonant mode on metallic superstrates. This mode is believed to arise from a coupled monopole-based resonance occurring between the side parts of the resonator. The results referred to here, exhibited two resonant locations, one from the finite sized metallic superstrate and another occurring due to the presence of the sensor. Although promising strain-sensitive results have been gathered both in simulation and in testing, the “on-metal” resonant response appears to exhibit some strong dependencies related to substrate loss tangent and others related to the size of the superstrate. Further study is needed on the “on-metal” the performance of this device, similar to that found in [53], but preliminary results suggest that it can operate as a viable sensor on these materials.

**Figure 13:**
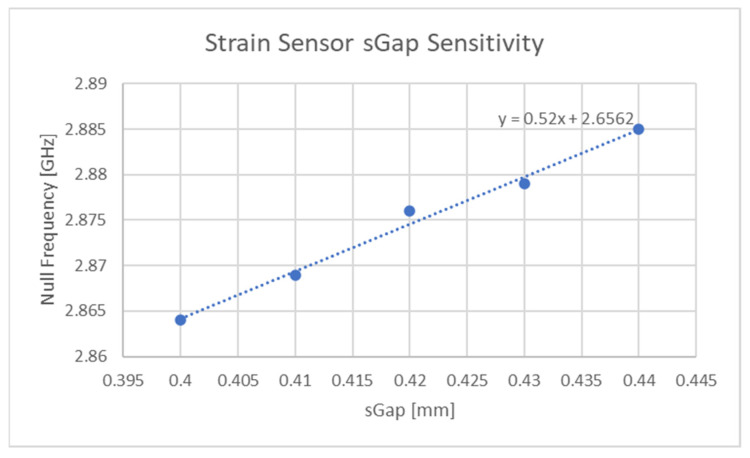
Rigid body strain sensitivity of resonator.

The dielectric response of the bare resonator against a change in “sGap“ is depicted in Figure 13. This figure is centred on the behaviour of the dip or null region of the curve seen in Figure 12 as this is the feature of the resonant response that moves with a change in the axial distance between the resonator parts. The curves presented in this figure had their meshing frequency finely tuned to their minimum frequency point such that the finest accuracy could be achieved. The “sGap” variable is varied between 0.4 mm to 0.44 mm, which corresponds to a strain of 0.23%.

**Figure 14:**
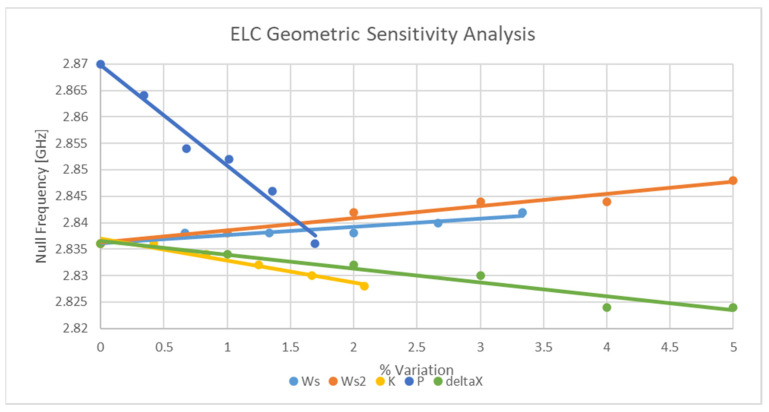
Sensitivity analysis of resonator geometry.

As the implementations of this sensor on relatively stiff substrates will result in other deformation mechanisms other than rigid body motion, further simulations are needed to assess these effects. The general sensitivity of each of the design parameters is depicted in Figure 14. Although some parameters are more sensitive than others, it can be concluded that other deformation mechanisms will cause changes in the resonant response of the device. Regarding the possible attenuation of the strain sensitivity of the device due to the cumulative effects of each geometric change, the aspect ratio of the sensor can be modified such that this possible effect is attenuated. 

#### 3.1.2. Physical Testing Results

**Figure 15:**
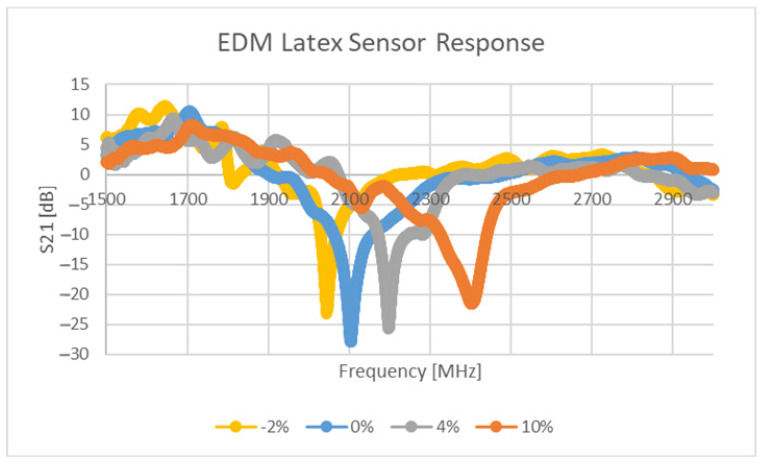
Response of sensor at two different stimulus levels.

**Figure 16:**
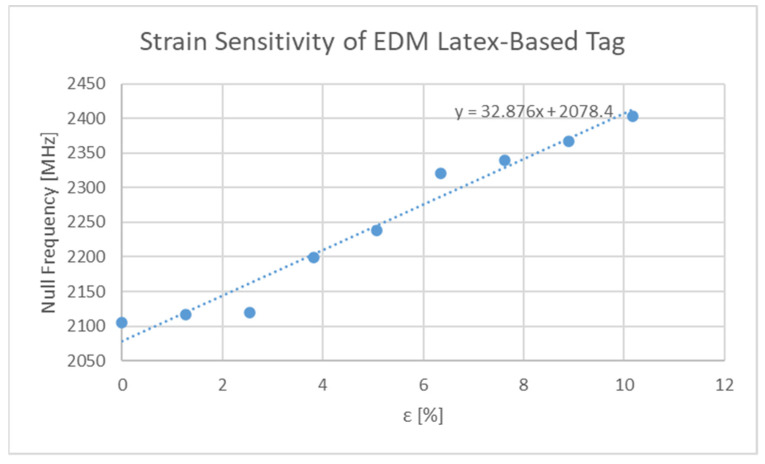
Sensitivity plot of physical sensor.

The response of the sensor depicted in Table 3 and Figure 3, for two different levels of deformation (0, 20%) are shown in Figure 15. Furthermore, the null of the sensor response against stimulus level is graphed in Figure 16. Values of S21 above 0 dB arise due to the background not including the test press and substrate during background subtraction tests. Clearly the sensor is strain sensitive, but the performance of the sensor appears weak below 3%. The authors believe that the stiffness of the resonator and the small distances between its constituent parts present a rigid region on the top surface which needs a significantly larger amount of stress to begin to significantly deform it. In this region, the direction of change in null frequency is of the same sign as that found in the larger dataset in Figure 16. Enhancing the sensitivity of the sensor in this region would require initialising the sensor with a larger “sGap” dimension or through the use of a reduced relative stiffness of the conductor to that of the substrate. The approximate sensitivity of this sensor is 32.8 MHz/%ε which corresponds to a gauge factor of 1.56.

Other physical test results corresponding to the other tags made with the cheaper conductor also exhibited strain sensitive characteristics. However, the magnitude of the dip observed with these designs was below 5 dB. One feature of interest of the performance of the other sensor designs is that the observed strain sensitivity was higher than that presented in the figure above. Further discussion of this will not be presented here but the presence of a thicker resonator forces a greater degree of rigid body motion and thus a greater strain sensitivity. Overall, it can be concluded from the existing sensor implementations, that the outlined sensor design is capable of operating as a strain sensor.

#### 3.1.3. Comparison with Other Works

Although the issues with a direct comparison between this work and that of others has been outlined earlier, it is still pertinent to present a basic performance comparison. Table 7 depicts the basic performance comparison of the results presented in Figure 16 above, against other works in the literature. 

From the data presented in Table 7 above, it is clear that several works have strain sensitivities exceeding that of this design. With that being said, the gauge factor of this work stands up well against many of those works whilst still being designed to support fabrication with printing technologies. Moreover, the implementation presented in this work has only received proof-of-concept testing and implementation. Furthermore, although this sensor has not been physically tested with a stiff substrate, the results of the EM analysis (see Figure 14) would suggest that this design should be sensitive to the other deformation mechanisms also.

### 3.2. Swelling and Orientation Analysis

#### 3.2.1. Investigation into Thermal Effects

**Figure 17:**
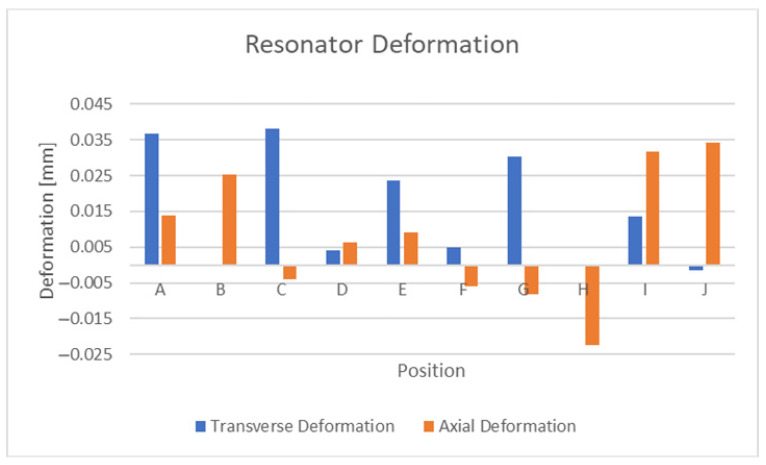
Thermal expansion deformations at 350 °C.

With the bottom of the substrate constrained by a free support, the axial deformation (0.33%) of the sensor and the effects of thermal expansion (350 °C) were simulated. The latter temperature was used in this analysis as other aerospace strain sensor systems have recorded this as an upper testing limit [19]. The resulting deformation of the top of the conductive surface is depicted in Table 7. Figure 17 graphically depicts the effect of thermal expansion at 350 °C with the bottom surface of the substrate in a fixed configuration. From the axial deformation results presented in Table 8, it is clear that a combination of rigid-body motion, bending and elastic deformation will occur with this substrate:conductor combination. The presence of bending and elastic deformation is evident from the fact that deformation of certain different positions vary to differing degrees after loading and unloading.

The axial deformation results presented in Table 7 show that the capacitance regions (position C to position I,J) along the perimeter of the resonator expand with increasing strain. Furthermore, it can be seen that the same can be said about the central capacitance (position G,H). One factor of interest is that the central capacitance capacitor plate experiences significant levels of bending. The thermal expansion results presented in the same table, reveal the significance of thermal expansion to the overall performance of the sensor, as its deformation magnitudes exceed that found in the axial deformation tests. The results obtained with the bottom surface of the substrate contained are significantly lower but are still comparable to the 0.33% ε axial tests.

**Figure 18:**
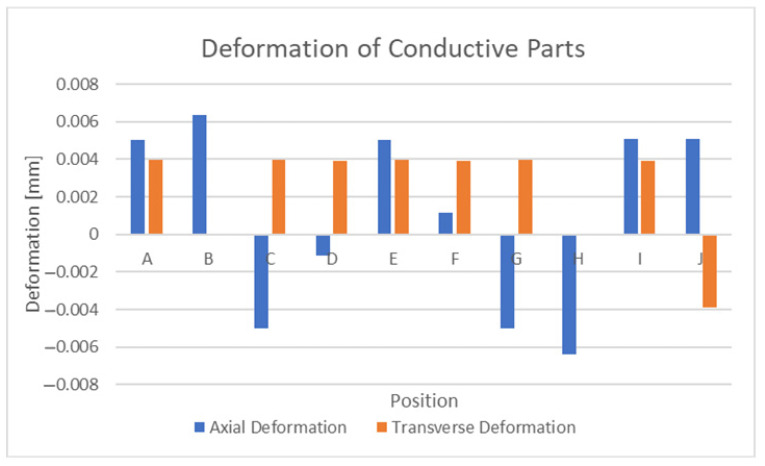
Deformation of isolated conductive elements at 350 °C.

The next question to ask was whether in an ideal case, where the substrate does not expand, will the swelling effects within the conductor parts be significant enough to disrupt successful strain sensing around 10 με. Figure 18 displays the results conductor swelling with the bottom surfaces of the conductors in a fixed configuration. The results presented in Figure 18 demonstrate that the conductor swelling occurred under these conditions with certain positions experiencing equivalent magnitudes of around 0.0417% ε (417 με) axial strain. The other point of interest in this discussion is that swelling causes expansive motion whereas uniaxial deformation, ignoring Poisson’s effect, causes deformation only in that particular direction. Clearly, the thermal expansion of the copper elements, which is dependent on the CTE value, appears significant enough to cause problems when attempting to sense strain within the small range of several thousand microstrain. 

#### 3.2.2. Investigation into Humidity Effects

**Figure 19:**
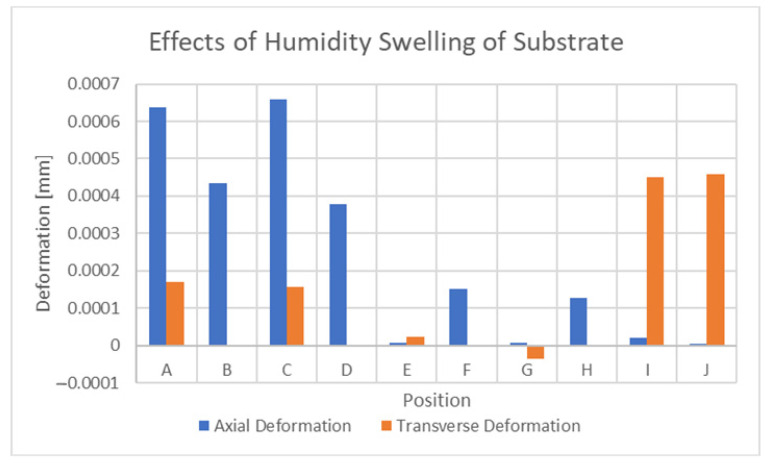
Effects of humidity-based swelling at 100% RH.

The effects of humidity on the sensor were slightly different than that of temperature. Figure 19 displays the results of humidity-based swelling at 100% relative humidity, with the bottom surface of the substrate fixed in place. Interestingly, the magnitudes of these deformations are much lower and also appear to have a dominant direction. This is most likely caused by the fact that the conductor is not swelling in this case but only experiences deformation through its contact region with the substrate. The axial deformation of this implementation has magnitudes (0.00066 mm) similar to that of 55 με.

#### 3.2.3. Substrate Design Results

**Figure 20:**
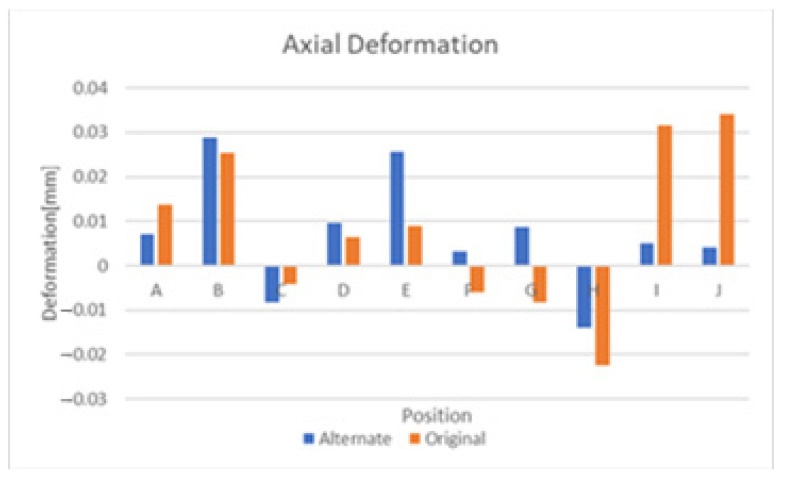
Axial deformation with symmetric substrate.

**Figure 21:**
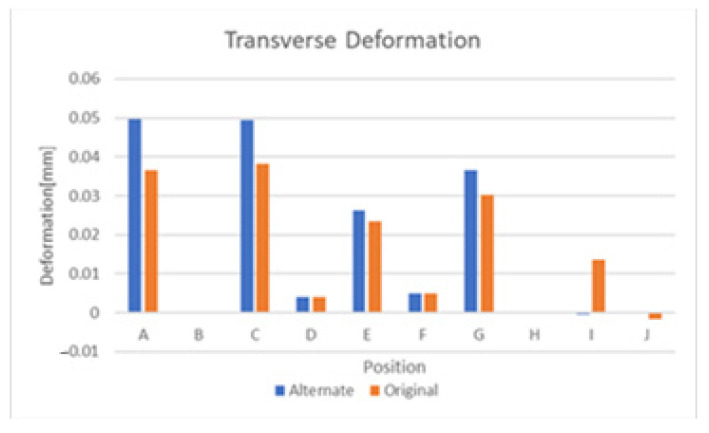
Transverse deformation with symmetric substrate.

From the previous section, it is apparent that significant levels of deformation can be attributed to thermal swelling and lesser but still significant levels also occur due to humidity-based swelling. As thermal swelling is by far the most significant of the two, this section will explore the effects of various substrate modifications that attempt to mitigate its effect. The symmetric substrate depicted in Figure 10 was explored first and the comparative results of this substrate and the original design are depicted in Figure 20 and Figure 21. These figures display results that the deformation of the sensor is largely similar, but some positions exhibit a significant improvement in deformation. Other positions exhibit an increase in deformation and the end conclusion of this substrate design is that it did not significantly reduce the negative effects of temperature-based swelling. Exploring the effects of thermal swelling as a function of temperature revealed that the deformations change in a linear fashion between 0 °C and 350 °C.

The other substrate design depicted in Figure 11 revealed some more promising results. Currently the efforts have worked to tune the deformation of position “C” to a minimum by varying the parameters outlined in Figure 11. Tuning of these parameters led to a 99.956% reduction in the axial deformation of that position. Testing of this design from 0–350 °C revealed that this deformation level did not vary linearly with temperature. Within this temperature range, the reduction in deformation of position “C” reached a minimum value of 99.213%. Further study is needed on this particular substrate design strategy, but an automated, iterative approach would appear to be the only way to tune the substrate geometric parameters such that the deformation of the entire resonator is minimised. The equivalent axial deformation at position “C” at its maximum is −30 με. This is still relatively large and attempts at modifying the substrate such that many positions experience a summed minimum level of deformation would surely exceed this value. Other design attempts made used copper guard rings on and around the top surface of the substrate to try and mitigate its expansion, but the results were unsatisfactory.

Overall, these results suggest that compensation for these effects within the sensor design, which were assumed to be the easiest to compensate for, may be too difficult to deal with within the design. Another important result is that two different stimuli, temperature and humidity can both give rise to swelling but not in the same manner. The effects of thermal swelling are a result of the swelling of two parts and the effects of humidity-based swelling are a result of the swelling of a single part. In order to determine the swelling effects on the sensor response, a reference sensor will be required. However, it is not immediately obvious if a single reference sensor can be developed that can sit nearby the strain sensor whose response can be decoupled from the total RCS response. The reason why this comment is made is that the conductors deform to a lesser degree during purely substrate swelling and thus rigid-body motion is the more dominant deformation mechanism under these circumstances. It is more likely than not that the strain sensitivity curve will itself be sensitive to changes in resonator geometry and thus, since humidity variations cause substrate swelling and temperature variations cause both substrate and resonator swelling, the two stimuli will cause different changes to the general sensor sensitivity. The problem with this finding is that a single reference sensor will give you a single value for the current swelling effects there are two variables for a single equation in this scenario and the two variables will most likely need to be known so that the appropriate change can be made to the sensitivity curve used in the strain value lookup procedure. Issues such as this may not be of critical importance in many strain sensing applications but reliable strain sensing below 100 με will most likely require these and other issues to be properly addressed.

#### 3.2.4. General Chipless RFID Tag/Sensor Orientation Challenges

**Figure 22:**
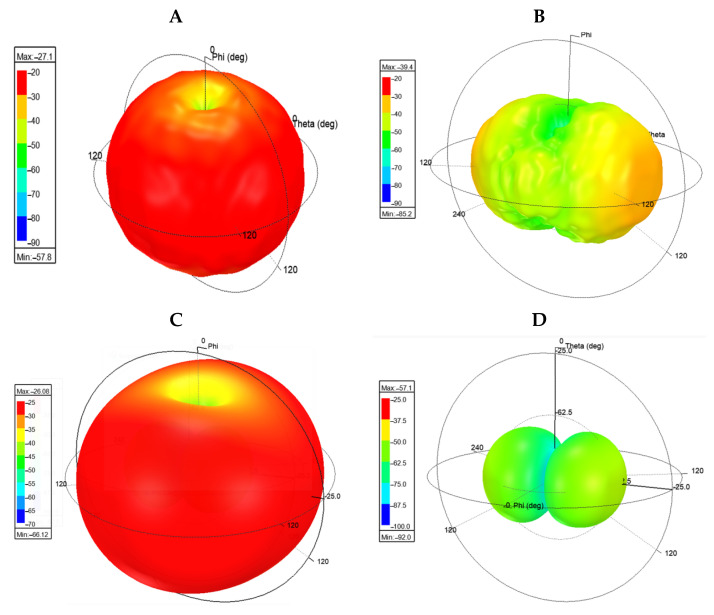
HFSS 3D electric field strength patterns of ELC-based resonators (images courtesy of ANSYS Inc). (**A**) Peak rE of sensor; (**B**) null rE of sensor; (**C**) peak rE of regular ELC; and (**D**) null rE of regular ELC.

Several works including [32,36,37] have briefly discussed the response characteristics of chipless RFID tags, depending on the orientation of the tag to the interrogating antennas. One possibility that is largely ignored is that some of these effects may include a variation of the null frequency of the tag. Such an issue is of critical importance for this sensor as it may need to be compensated for if the magnitude of variation is significant. Figure 22A,B depict the approximated peak and null 3D electric field strength (rE) of the sensor. Similarly, Figure 22C,D depict the peak and null 3D electric field strength (rE) of an ELC resonator. Clearly, the null rE does not appear to be a scaled down version of the peak rE in either of the chipless RFID designs. Thus, even if the null is consistent at all angles of interrogation in these designs, the width of the resonant region will be orientation dependent as the rate of change of the electric field strength as a function of frequency is clearly orientation dependent. This result has ramifications for sensors of this type that make use of the Q-factor of the resonator to encode stimulus information.

**Figure 23:**
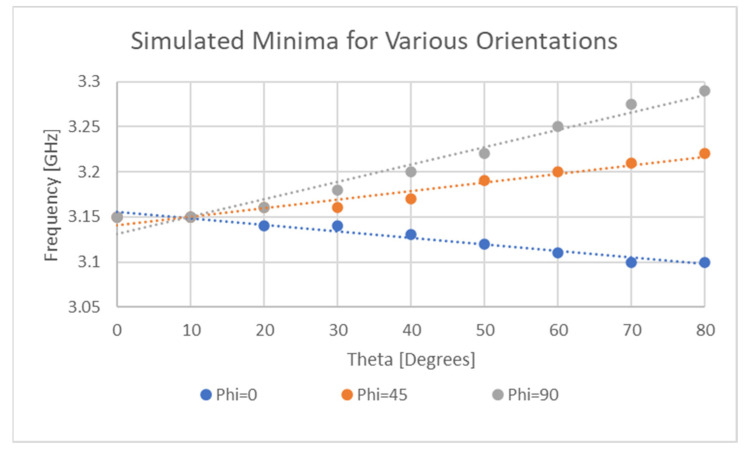
Orientation sensitivity of sensor resonant response.

**Figure 24:**
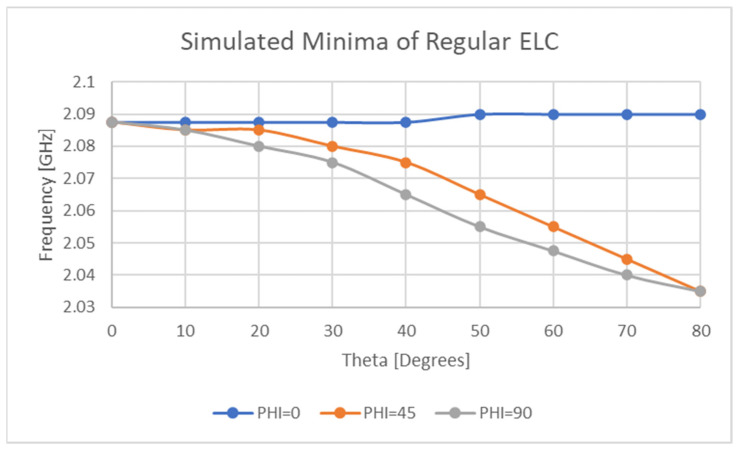
Orientation sensitivity of ELC resonant response.

Figure 23 and Figure 24 reveal the variation in null frequency against theta for the sensor and the standard ELC resonator for three different alpha angles. This result is of critical importance as it displays that for two different chipless RFID tag/sensor designs, the location of the null frequencies is more likely than not, to be highly orientation dependent. Basic physical testing was also performed at distances of between 1λ and 3λ from the sensor which revealed a change in 28.8 MHz by changing the Rx antenna orientation from a theta angle of 0° to 30°.

The results present in this section demonstrate that this sensor and another popular chipless RFID tag will exhibit weak orientation sensitivities. Simulation results exhibit variations of up to 150 MHz which is a significant result for this application however, as the strain sensitivity of the implemented sensor was only 36 MHz per percent strain. 

#### 3.2.5. Further Discussion

From the analysis carried out in the previous subsections, it is clear that the effects of swelling and sensor orientation will have a significant impact on the resonant response of the strain sensor. Although many of the effects outlined in the Introduction may impact the resonant response, the two effects explored in this work would appear to be largely ignored in the existing literature. An obvious critique of this analysis is that the results are specific to the strain sensor outlined in this work and that such a scenario may not arise with other designs. The results of expansion of the isolated conductive elements are significantly lower than that of the total assembly, despite the fact that the CTE/CHE and stiffness of the substrate are significantly lower. This would suggest that despite the conductive elements being significantly stiffer and more resistant to expansion, the properties of the substrate would appear to be the dominant contributor to swelling. Such a suggestion inherently leads to the idea that the resonator geometry will not significantly impact the effects of sensor swelling. From the analysis of sensor orientation, it would appear that Q-factor and null frequency of the sensor response will vary with orientation. In scenarios where the environment is static, initial testing should be performed under known loading conditions to allow for calibration of the sensor response.

## 4. Conclusions

### 4.1. Overall Conclusions

This work set out to develop a novel chipless RFID strain sensor, which has been successfully achieved. The strain sensor developed in this work exhibits an impressive gauge factor, exceeding unity, and there is sufficient evidence that it should operate successfully with a variety of different conductor-substrate material combinations. More generally, this paper has attempted to dismantle the current strategy used in the development of chipless RFID strain sensors that has largely resulted in the comparison of substrate materials.

The exploration of humidity and thermally induced swelling has revealed that these effects are significant enough to hinder the sensing of strain within a resolution of 10 με. Furthermore, given the fact that swelling is the only effect considered in this analysis, it seems inevitable that the general issue of cross sensitivity is a much larger issue than described within this small body of work. 

A basic exploration of the orientation-dependent resonant behaviour of this sensor and the basic ELC resonator is performed in this work, largely through the use of simulations. More generally, issues of this type may be of little concern to the chipless RFID tag community but appear to be significant enough in the area of chipless RFID-based sensing. Arguably, this issue is of secondary performance to the goal of developing a proof-of-concept sensor design, but as some point in the development of chipless RFID sensor technology this particular issue will need to be explored. As mentioned earlier in the document, the magnitude of this issue will be design-specific and thus future sensors of this type should attempt to describe the orientation-based limitations of the response recorded during idealistic testing.

### 4.2. Future Work

This work has put forward a modified ELC resonator, largely because of its suitability for supporting large deformations and its high sensitivity. Other designs exist which boast other advantages such as polarization insensitivity and/or strong operation on conducting superstrates. The main reason why a single design should be pushed forward is that it would allow for subsequent focused exploration of other challenges around strain sensing, such as various cross sensitivities, orientation limitations, and complete in situ fabrication. The effects of environmental stimuli such as temperature and humidity have been explored via the use of simulation modelling. Reasons as to why physical testing has been ignored in this analysis is that material properties can vary significantly with fabrication method and fabrication conditions. The future aims include of this overall work are as follows:

- Proof of concept strain sensing below 0.2% with this or an enhanced resonator design on a stiff substrate. This sensor should make use of the other deformation mechanisms that this work largely avoids;
- Sensor fabrication using an established, in-situ fabrication method that will support consistent electrical, thermal and mechanical sensor properties. Then, reliable physical testing should be performed with varying environmental conditions such as humidity and temperature;
- Full characterization of the performance of this sensor on dielectric and conducting superstrates below 0.2% strain;
- Exploration of design methods to mitigate/compensate for the possible transverse strain sensitivity of this current sensor design.

## Figures and Tables

**Table 1:** Resonator Sensitivity List.

Variable Name	Known Dependencies	Comment
Axial Strain	Axial deformation, Transverse strain (due to Poisson’s effect), material models	This variable is designed to be the dominant contributing variable to the sensor response. Where this is not possible, compensation will be required
Transverse Strain	Transverse deformation, substrate transverse expansion, conductor transverse expansion, material models	This variable should be mitigated against within the design or through compensation within the overall sensor implementation, as seen in Reference [11]
Substrate Expansion/Contraction	Thermal expansion, humidity-based swelling	Polyimides and other substrate materials of interest suffer from significant levels of humidity and/or thermal-based expansion [20,21]
Conductor Expansion/Contraction/Material Loss	Thermal expansion, corrosion, material models	This parameter is perhaps one of the more difficult variables to mitigate against. This variable can be reversable or irreversible as corrosion and creep can cause permanent expansion/contraction.
Conductor Resistance	Temperature, corrosion	Conductor resistance influences the Q-factor of chipless RFID tags. Certain resonant elements will also exhibit changes in null frequency. Corrosion could result in a complex change in resistance, caused by material loss and by surface oxidation
Structural material Models	Temperature, pressure, humidity	These models vary from simple isotropic elasticity models to more complex models that include effects such as creep. Most if not all these models contain properties that are sensitive to temperature [22,23,24] and other environmental parameters [25]
Dielectric Material Model	Temperature, humidity, pressure	Properties described by this model consist of dielectric constant(permittivity) and loss tangent. These parameters can be highly sensitive to environmental effects within a variety of dielectric materials [26,27]

**Table 2:** Ecoflex tag dimensions.

Variable	Value [mm]	Variable	Value [mm]
S2	50	Ws	2
S	24	Ws2	2
sGap	1	K	19
H_ecoflex	5.5	P	14
H_glove	<0.2	deltaX	10

**Table 3:** Latex EDM tag dimensions.

Variable	Value [mm]	Variable	Value [mm]
S2	76	Ws	2
S	24	Ws2	2
sGap	1	K	19
H_substrate	3	P	14
H_resonator	0.3	deltaX	10

**Table 4:** Ecoflex EDM tag dimensions.

Variable	Value [mm]	Variable	Value [mm]
S2	50	Ws	2.5
S	30	Ws2	2.5
sGap	<0.2	K	17
H	5	P	15
H_resonator	<0.3	deltaX	5

**Table 5:** Thermal and mechanical coefficients.

Material	Young’s Modulus	Poisson’s Ratio	Coefficient of Thermal Expansion	Thermal Conductivity
Polyimide [52,53]	2.5 GPa	0.34	0.0001 C^−1^	0.12 Wm^−1^ C^−1^
Copper	125 GPa	0.345	0.0000168 C^−1^	385 Wm^−1^ C^−1^

**Table 6:** FEA sensor specifications.

Variable	Value [mm]	Variable	Value [mm]
S2	40	Ws	2
S	34	Ws2	3
sGap	0.4	K	12
H	0.5	P	6
H_resonator	0.05	deltaX	−10

**Table 7:** FEA sensor specifications.

Publication	Base Frequency [MHz]	Sensitivity [MHz/%ε]	Max Tested Stimulus [%]	Gauge Factor	Year
This Work	2100	32.88	10	1.57	2021
[11]	1550	−14	25	0.9	2020
[8]	1610	8.05	4	0.5	2014
[12]	1530	−13.68	0.05	0.89	2012
[7]	12,250	51.48	0.2	0.42	2009
[9]	860	−1.2	50	0.14	2019
[15]	3300	85	0.9	2.58	2013
[14]	2900	36.56	1.65	1.26	2011

**Table 8:** Deformation effects on ELC sensor.

Setup	A	B	C	D	E	F	G	H	I	J
0.05 mm Axial Def.	0.0246	0.0234	0.0249	0.022	0.055	0.014	0.0058	0.0128	0.0039	0.0053
350 °C Thermal Def.	0.074	0.0728	0.0576	0.055	0.020	0.023	0.032	0.0481	0.0515	0.0498

## Data Availability

Data available upon request and/or available on ResearchGate, accompanying details of this publication.

## Abbreviations

ELC	Electric LC
AgNP	Silver nanoparticle
MWCNT	Multi Walled Carbon Nanotube
MLA	Meander Line Antenna
SRR	Split Ring Resonator
MUT	Material Under Test
EM	Electromagnetic
RCS	Radar Cross Section
REP	Resonant Electromagnetic Particle
EDM	Electro-Discharge Machining
FEA	Finite Element Analysis
CTE	Coefficient of Thermal Expansion
CHE	Coefficient of Hygroscopic Expansion
